# The Consumption of Dietary Antioxidant Vitamins Modifies the Risk of Obesity among Korean Men with Short Sleep Duration

**DOI:** 10.3390/nu9070780

**Published:** 2017-07-20

**Authors:** Miae Doo, Yangha Kim

**Affiliations:** Department of Nutritional Science and Food Management, Ewha Womans University, Ewhayeodae-gil, Seodaemun-gu, Seoul 03760, Korea; miae_doo@ewha.ac.kr

**Keywords:** antioxidant, Korean Genome and Epidemiology Study, sleep duration, obesity

## Abstract

Short sleep duration has been reported to be associated with various health problems. This study examined the influence of sleep duration on the odds of being obese in relation to the consumption of dietary antioxidant vitamins among 3941 Korean men between 40 and 69 years of age. After adjusting for age, education, household income, marital status, insomnia, smoking and drinking status, participants with short sleep duration (<6 h) had significantly higher body mass index (*p* = 0.005), body fat mass (*p* = 0.010), body fat percentage (*p* = 0.021), waist circumference (*p* = 0.029), as well as the odds ratio (OR) of risk of obesity [OR (95% CI) = 1.467 (1.282–1.678)], compared to participants with optimal sleep duration (≥7 h). Short sleepers with a low consumption of dietary antioxidant vitamins had a higher risk of obesity than those with a high consumption of dietary antioxidant vitamins; however, this relationship did not hold among those with optimal sleep duration. Although a causal relationship among sleep-related variables could not be definitively demonstrated because of this study’s cross-sectional design, our results suggested that the increased risk of obesity associated with short sleep duration may be modified by the consumption of dietary antioxidant vitamins.

## 1. Introduction

Sufficient sleep duration is one of the most important factors for maintaining a healthy lifestyle. Sleep duration is inversely related to the prevalence of chronic diseases, including metabolic syndrome (MS), type 2 diabetes mellitus, dyslipidemia, and cardiovascular diseases [1,2]. Additionally, individuals who sleep for an insufficient duration showed an increased risk of obesity, which is related to differences in dietary consumption [3].

Diet is one of the potential factors for preventing or controlling obesity. Previous studies [4,5,6] have reported that obesity is probably related to less intake of dietary micronutrients, which is explained by a decline in dietary quality [4,7]. For example, an increase in obesity is associated with unhealthy dietary patterns, such as the consumption of a diet high in carbohydrates, fat, simple sugars and sweet beverages, whereas a protective effect against obesity is associated with the consumption of a diet high in fruits and vegetables [8,9,10]. A diet rich in vegetables and fruits has been positively associated with the consumption of antioxidant vitamins [8]. Epidemiological studies [10,11] have reported that the Mediterranean diet, which contains an adequate amount of antioxidant vitamins, such as β-carotene, vitamin C, and vitamin E, as well as various important minerals, is inversely associated with obesity-related variables.

Although the association of sleep duration with obesity [1,2,3] or the association of dietary antioxidant vitamin with obesity [4,5,8,9,10,11] is well established, the alteration of these associations by consumption of dietary antioxidant vitamins has not been clearly elucidated. Therefore, this study examined how the effect of sleep duration on the risk of obesity is modified via the consumption of dietary antioxidant vitamins among Korean men who participated in the Korean Genome and Epidemiology Study (KoGES).

## 2. Participants and Methods

### 2.1. Study Design and Participants Selection

Data from 3941 Korean men aged between 40 and 69 years who participated in the KoGES were used. The KoGES is an ongoing community-based cohort study that began in 2001 that was performed to investigate chronic diseases such as diabetes mellitus, hypertension, osteoporosis, obesity and MS among 10,038 Koreans aged 40–69 years old (5020 and 5018 from the urban Ansan and rural Ansung areas, respectively). Detailed information on the study design of the KoGES has been reported elsewhere [12]. Of the participants in the KoGES, participants with a dietary energy consumption of ≤800 kcal or ≥4000 kcal were excluded. In addition, participants with inadequate and missing data concerning their sleep duration and anthropometric variables were excluded from this study. The Institutional Review Board of the Korea Centers for Disease Control and Prevention approved the study protocol, and all participants provided written informed consent.

### 2.2. Data Collection

General characteristics, sleep-related variables, anthropometric measurements, blood pressure, blood biochemical variables, sleep-related variables, and dietary antioxidant vitamin consumption were collected using the KoGES data. Anthropometric measurements, blood pressure and blood biochemical variables were collected by direct measurement. Trained specialists collected data related to general characteristics, sleep-related variables and dietary consumption using standardized questionnaires and interviewers.

Anthropometric measurements, such as height, body weight, and waist circumference (WC) were measured using a standardized procedure. Body mass index (BMI) was calculated by dividing the participant’s weight in kilograms by height in meters squared. According to the World Health Organization (WHO) Asia-Pacific criteria for obesity [13], obesity was defined as a BMI of ≥25 kg/m^2^. Body composition variables such as lean body mass (LBM), body fat mass (BFM), and body fat percentage (P_BF) were analyzed using In-body 3.0 (Biospace Co., Ltd., Seoul, Korea). Blood pressures was measured using a mercury manometer with the participants in a sitting position after 5 min of rest. Systolic and diastolic blood pressures were recorded as the average of three readings. Blood samples were collected after an overnight fast to analyze biochemical measurements. Fasting glucose (FG), triglycerides (TGs), total cholesterol (TC), and high-density lipoprotein cholesterol (HDL-C) were measured using a Hitachi automatic analyzer 7600 (Hitachi, Tokyo, Japan).

General characteristics included demographic characteristics, socioeconomic status, and smoking and drinking status. Socioeconomic status included education level, monthly household income and marital status. Sleep duration was analyzed using a questionnaire that assessed participants’ usual sleeping hours per day. Participants were divided into two categories: ≤6 h/day (short sleep duration) and ≥7 h/day (optimal sleep duration), because a sleep duration of 7–8 h a day has been recommend as an optimal time for sleeping in previous studies [14,15]. Insomnia was defined based on the presence of 4 symptoms over the past month: difficulty initiating sleep, difficulty maintaining sleep, early morning awakenings and non-restorative sleep. The consumption of dietary antioxidant vitamins such as vitamin A, retinol, carotene, vitamin C and vitamin E was assessed using a semi-quantitative food frequency questionnaire (SQ-FFQ) developed and validated for the KoGES [16]. This questionnaire consisted of 103 food items, 3 serving sizes, and 9 consumption frequency categories that were combined into the 23 nutrients used in the Korean food composition table. The dietary antioxidant vitamins were expressed in μg per 1000 kcal total energy consumption. Participants were divided into 2 groups according to their median level of consumption of dietary antioxidant vitamins (vitamin A: 238.62 μg RAE/1000 kcal, retinol: 30.00 μg/1000 kcal, carotene: 1165.89 μg/1000 kcal, vitamin C: 52.27 mg/1000 kcal and vitamin E: 4.50 μg/1000 kcal, respectively).

### 2.3. Statistical Analysis

Before the data analyses were conducted, all continuous variables were examined for normal distributions, and logarithmic transformations were performed on skewed variables such as FG and TGs. The data are presented as mean ± standard errors of the mean (SEMs). As a crude model, categorical variables were assessed using Pearson’s chi-square test, whereas continuous variables were assessed using independent-samples *t*-tests. Generalized linear models were analyzed to determine the effect of sleep duration on the obesity-related variables including anthropometric measurements, blood pressure, biochemical variables, and dietary antioxidant vitamin consumption after adjusting for covariates. To limit confounding effects, the covariates were adjusted for participant age (years), education level (≤middle school/≥high school), monthly household income (low/high), marital status (no/yes), presence of insomnia (no/yes), current smoking status (no/yes), and alcohol consumption status (no/yes). A multinomial logistic regression model adjusted for covariates was used to analyze the effect of the interaction between sleep duration and dietary consumption on obesity. The odds ratios (ORs) and 95% confidence intervals (CIs) for obesity were estimated for each consumption level of dietary antioxidant vitamins and a sleep duration of ≥7 h per day. A *p*-value of < 0.005 was regarded as significant. All analyses were conducted using SPSS (version 21.0; IBM Corporation, Armonk, NY, USA) for Windows. 

## 3. Results

The General characteristics of the 3941 Korean men classified by sleep duration are shown in Table 1. The average ages of participants with short sleep and optimal sleep durations were 51.17 and 51.88 years, respectively (*p* = 0.012). Educational level (*p* < 0.001), household income (*p* < 0.001), insomnia diagnosis (*p* < 0.001), and smoking status (*p* = 0.005) differed by sleep duration. However, marital status and drinking status did not differ by sleep duration. 

The men with short sleep duration were shown to be at a significantly higher risk of obesity according to the crude and adjusted models (Table 2). In the crude model, BMI (24.47 ± 2.90 kg/m^2^ vs. 24.16 ± 2.92 kg/m^2^, *p* = 0.001), BFM (15.51 ± 0.13 kg vs. 15.01 ± 0.11 kg, *p* = 0.002), P_BF (22.13 ± 4.80 kg vs. 21.69 ± 4.93, *p* = 0.013), TC (194.00 ± 36.50 mg/dL vs. 191.55 ± 36.41 mg/dL, *p* = 0.039), and LDL-C (116.93 ± 33.34 mg/dL vs. 114.21 ± 33.59 mg/dL, *p* = 0.015) were significantly higher in participants with short duration than in those with optimal duration of sleep. In addition, LBM (53.29 ± 0.16 kg vs. 53.03 ± 0.14, *p* = 0.041) and WC (83.99 ± 7.64 cm vs. 83.51 ± 7.57 cm, *p* = 0.049) were marginally associated with sleep duration according to the crude model. However, BMI (*p* = 0.005), BFM (*p* = 0.010), P_BF (*p* = 0.021), and WC (*p* = 0.029) were associated with sleep duration in the covariate-adjusted model.

When the effect of sleep duration was analyzed with regard to dietary antioxidant vitamin consumption, the consumptions of dietary retinol (*p* = 0.024) and vitamin E (*p* = 0.032) differed according to sleep duration in the crude model (Table 3). However, the covariate-adjusted model did not reveal significant differences with regard to the consumption of any of the dietary antioxidant vitamins and sleep duration. 

**Figure 1:**
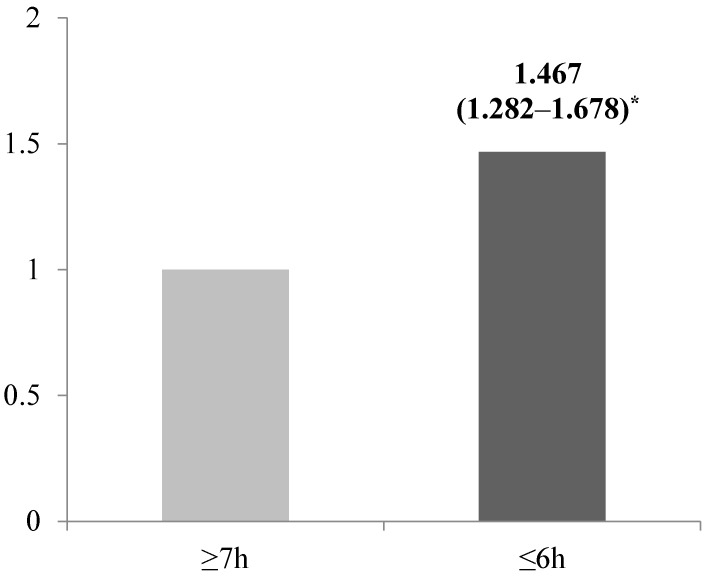
Adjusted odds ratio for obesity risk according to sleep duration. Obesity was defined as BMI ≥ 25.0 kg/m^2^ by the International Obesity Task Force (IOTF) Asia-Pacific region standard. * OR (95% CIs) and *p*-values as a reference for sleep duration ≥7 h per day using a multivariate logistic regression after adjusting for age, education level, household income, marital status, insomnia, and smoking and drinking status (*p* < 0.001).

To analyze whether sleep duration affected the odds of obesity, a logistic regression model was examined after adjusting for the covariates in Figure 1. A significant association was found between sleep duration and the odds of being obese (*p* < 0.001). The odds of obesity were 1.467-fold (95% CIs = 1.282–1.678) greater among participants who slept for short durations compared with those who slept for optimal durations; this difference was significant.

To assess the combined effect of dietary antioxidant vitamin consumption and sleep duration on the odds of being obese, a multinomial logistic regression analysis was performed after adjusting for covariates (Table 4). Using high consumption of dietary antioxidants and a sleep duration of ≥7 h per day as a reference, the participants who slept ≥7 h per day exhibited no difference in the odds of being obese, regardless of dietary antioxidant vitamin consumption. However, a significant difference was found in the odds of being obese among participants with short durations. In other words, among participants who slept ≤6 h per day, the odds of being obese showed a significantly increasing trend with a low consumption of dietary antioxidants compared with a high consumption of dietary antioxidant vitamins (*p* for trend < 0.001 for all vitamins). Among men who slept ≤6 h per day, the odds of being obese were 1.429-fold (95% CI = 1.177–1.736) higher among participants with a high consumption of dietary vitamin A, whereas the odds of being obese were 1.503-fold (95% CI = 1.245–1.815) higher among participants with a low consumption of dietary vitamin A. These findings showed similar results with regard to the consumption of dietary retinol, carotene, vitamin C, and vitamin E. Among participants with short sleep durations, the ORs associated with obesity were 1.334 (95% CI = 1.099–1.619) in participants with a high consumption of retinol, 1.330 (95% CI = 1.096–1.614) in those with a high consumption of carotene, 1.491 (95% CI = 1.246–1.784) in those with a high consumption of vitamin C, and 1.475 (95% CI = 1.218–1.787) in those with a high consumption of vitamin E. However, the ORs increased to 1.347 (95% CI = 1.116–1.625) in participants with a low consumption of retinol, 1.511 (95% CI = 1.252–1.825) in those with a low consumption of carotene, 1.616 (95% CI = 1.331–1.963) in those with a low consumption of vitamin C, and 1.675 (95% CI = 1.388–2.022) in those with a low consumption of vitamin E. Conversely, no significant differences in the odds of being obese were observed in terms of dietary consumption of retinol, carotene, vitamin C, and vitamin E among participants who slept ≥7 h per day.

## 4. Discussion

This study used data from the KoGES, a large community-based cohort study, to determine whether the consumption of dietary antioxidant vitamins may modulate the risk of obesity related to a short sleep duration. Not only obesity-related variables but also the risks of being obese were found to be associated with a short sleep duration among 3941 Korean men aged 40–69 years in a model that adjusted for age, education, household income, marital status, insomnia, smoking, and drinking status. Additionally, there was a combined effect of sleep duration and dietary antioxidant vitamin consumption on the odds of being obese: participants with a short sleep duration and low dietary antioxidant vitamin consumption exhibited an increasing trend in the odds of being obese compared with those with a high consumption of dietary antioxidant vitamins, whereas the participants with an optimal sleep duration did not have increased odds of being obese, regardless of dietary antioxidant vitamin consumption.

The results of this study were consistent with those of previous studies [1,2,3]; they generally established an influence of short sleep duration on obesity. Men who slept for short durations showed higher BMIs, BFMs, P_BFs, as well as a greater risk of being obese compared with those who slept for optimal durations in the covariates-adjusted model. Although the direction of the causal relationship between sleep duration and obesity-related variables could not be determined because of the cross-sectional study design, these results might be explained by the observations of our previous studies [2,3]. Specifically, a short sleep duration might support increased appetite, subjective hunger, and dietary consumption because the level of ghrelin, an appetite stimulant, was elevated while the level of leptin, a satiety signal, was decreased, which is related to increases in obesity-related variables and the risk of obesity [17,18].

The consumption of dietary antioxidant vitamins was not associated with sleep duration in this study. However, this finding is inconsistent with previous studies [7,19] reporting a relationship between sleep duration and healthy dietary habits. One potential reason for that relationship, which was proposed by Grandner et al. [7], is that a short sleep duration may lead to decreased dietary quality, reduced food diversity or decreased consumption of healthy foods, such as vegetables and fruits, which are considered relatively rich in antioxidant vitamins. 

Although no differences in dietary antioxidant vitamin consumption associated with sleep duration or the odds of being obese via dietary antioxidant vitamin consumption were observed, interestingly, after adjusting for covariates, a combined effect of the consumption of dietary antioxidant vitamins, such as vitamin A, retinol, carotene, vitamin C, and vitamin E, on sleep duration was observed with respect to the odds of being obese. The odds of being obese among participants who consumed less antioxidant vitamins, especially among short sleepers, increased compared with those who consumed more dietary antioxidant vitamins. These findings suggested that those with a short duration of sleep require dietary antioxidant vitamin consumption with respect to becoming obese. 

The main limitation of this study is that due to its cross-sectional design, it cannot provide a causal explanation of the results; thus, the findings should be interpreted and considered carefully. Future studies are needed to determine the causes and precedents of the associations between sleep duration and dietary antioxidant vitamin consumption with regard to obesity. In addition, because the consumption of antioxidant vitamins were influenced with dietary vegetables and fruits as well as supplements containing the identified antioxidants, future studies should be considered with more detailed data of dietary antioxidant vitamins and biochemical data concerning serum antioxidant vitamins.

In summary, this study used data from the KoGES to demonstrate that a short sleep duration is linked to obesity-related variables and the risk of obesity among Korean men. In addition, short sleepers with a low consumption of dietary antioxidant vitamins showed an increased risk of obesity compared with those with a high consumption of dietary antioxidant vitamins; however, this relationship did not hold among those with optimal sleep duration. These findings suggested that the increased odds of being obese based on a short sleep duration could potentially be modulated by a higher consumption of dietary antioxidant vitamins.

## Figures and Tables

**Table 1:** General characteristics according to sleep duration in Korean men.

General Characteristics		≥7 h/Day (*n* = 2360)	≤6 h/Day (*n* = 1581)	*p*-Value *
Age (years)		51.88 ± 8.84	51.17 ± 8.56	0.012
Education level	≤9 years	44.2	36.3	<0.001
	≥9 years	55.8	63.7	
Household income	Low	43.4	37.1	<0.001
	High	56.6	62.9	
Marriage status	No	4.0	4.1	0.471
	Yes	96.0	95.9	
Insomnia diagnosis	No	91.9	84.3	<0.001
	Yes	8.1	15.7	
Drinking status	No	18.1	18.7	0.347
	Yes	81.9	81.3	
Smoking status	No	49.2	53.4	0.005
	Yes	50.8	46.6	

Values are means ± SEM or %; * *p*-values between sleep duration using *x*^2^-test or *t*-test.

**Table 2:** Obesity-related variable according to sleep duration in Korean men.

Obesity-Related Variables	≥7 h/Day (*n* = 2360)	≤6 h/Day (*n* = 1581)	*p*-Value *	*p*-Value **
BMI (kg/m^2^)	24.16 ± 2.92	24.47 ± 2.90	0.001	0.005
LBM (kg)	53.03 ± 0.14	53.29 ± 0.16	0.041	0.277
BFM (kg)	15.01 ± 0.11	15.51 ± 0.13	0.002	0.010
P_BF (%)	21.69 ± 4.93	22.13 ± 4.80	0.013	0.021
WC (cm)	83.51 ± 7.57	83.99 ± 7.64	0.049	0.029
SBP (mmHg)	117.40 ± 16.46	117.12 ± 16.63	0.593	0.405
DBP (mmHg)	76.33 ± 11.03	76.08 ± 11.46	0.501	0.766
FG (mg/dL)	90.26 ± 23.74	91.07 ± 24.24	0.280	0.344
TG (mg/dL)	179.72 ± 126.91	176.05 ± 110.10	0.674	0.189
TC (mg/dL)	191.55 ± 36.41	194.00 ± 36.50	0.039	0.460
HDL-C (mg/dL)	43.57 ± 10.01	43.71 ± 9.83	0.663	0.355
LDL-C (mg/dL)	114.21 ± 33.59	116.93 ± 33.34	0.015	0.308

BMI, Body mass index; LBM, Lean body mass; BFM, Body fat mass; P_BF, Percentage body fat; WC, Waist circumference; SBP, Systolic blood pressure; DBP, Diastolic blood pressure; FG, Fasting glucose; TG, Triglycerides; TC, Total cholesterol; HDL-C, High density lipoprotein cholesterol; LDL-C, Low density lipoprotein cholesterol; Values are means ± SEM; * *p*-values between sleep duration using the *x*^2^-test; ** *p*-values between sleep duration using a general linear model after adjusting for age, education level, monthly household income, marital status, insomnia, and smoking and drinking status.

**Table 3:** Dietary antioxidant consumption according to sleep duration in Korean men.

Dietary Consumption	≥7 h/Day (*n* = 2360)	≤6 h/Day (*n* = 1581)	*p*-Value *	*p*-Value **
Energy (kcal)	1995.55 ± 544.60	2008.54 ± 527.58	0.457	0.702
Vitamin A (μg RAE/1000 kcal)	274.89 ± 160.93	273.28 ± 155.20	0.754	0.561
Retinol (μg/1000 kcal)	33.09 ± 24.07	34.87 ± 24.89	0.024	0.221
Carotene (μg/1000 kcal)	1422.66 ± 1006.92	1400.66 ± 968.21	0.492	0.430
Vitamin C (mg/1000 kcal)	58.56 ± 30.65	58.98 ± 30.27	0.670	0.938
Vitamin E (μg/1000 kcal)	4.61 ± 1.35	4.70 ± 1.34	0.032	0.242

Values are means ± SEM; * *p*-values between sleep duration using the *x*^2^-test; ** *p*-values between sleep duration using a general linear model after adjusting for age, education level, monthly household income, marital status, insomnia, and smoking and drinking status.

**Table 4:** Adjusted odds ratio for obesity according to dietary antioxidant consumption and sleep duration among Korean men.

Dietary Consumption	Sleep Duration		*p*-Trend
	≥7 h/Day	≤6 h/Day	
Vitamin A			<0.001
High	Reference	1.429 (1.177–1.736)	
Low	1.002 (0.843–1.190)	1.503 (1.245-1.815)	
Retinol			<0.001
High	Reference	1.334 (1.099–1.619)	
Low	0.838 (0.704–0.997)	1.347 (1.116–1.625)	
Carotene			<0.001
High	Reference	1.330 (1.096–1.614)	
Low	0.941 (0.793–1.118)	1.511 (1.252–1.825)	
Vitamin C			<0.001
High	Reference	1.491 (1.246–1.784)	
Low	1.118 (0.941–1.329)	1.616 (1.331–1.963)	
Vitamin E			<0.001
High	Reference	1.475 (1.218–1.787)	
Low	1.156 (0.973–1.374)	1.675 (1.388–2.022)	

OR (95% CI), odds ratio (95% confidence interval). Obesity was defined as BMI ≥ 25.0 kg/m^2^ by the International Obesity Task Force (IOTF) Asia-Pacific region standard. The consumption of dietary antioxidant vitamins were categorized as low and high according to their median level. OR (95% CIs) and *p*-values as a reference for the high-level consumption of dietary antioxidants and sleep duration ≥7 h per day using a multivariate logistic regression after adjusting for age, education level, household income, marital status, insomnia, and smoking and drinking status.
